# Hand preference across the lifespan: effects of end-goal, task nature, and object location

**DOI:** 10.3389/fpsyg.2014.01579

**Published:** 2015-01-20

**Authors:** Claudia L. R. Gonzalez, Jason W. Flindall, Kayla D. Stone

**Affiliations:** The Brain in Action Laboratory, Department of Kinesiology, University of LethbridgeLethbridge, AB, Canada

**Keywords:** grasp-to-eat, visuomotor control, left hemisphere, action intent, development, senescence

## Abstract

In the present study we investigate age-related changes in hand preference for grasping and the influence of task demands on such preference. Children (2–11), young-adults (17–28) and older-adults (57–90) were examined in a grasp-to-eat and a grasp-to-construct task. The end-goal of these tasks was different (eat vs. construct) as was the nature of the task (unimanual vs. bimanual). In both tasks, ipsilateral and contralateral grasps were analyzed. Results showed a right-hand preference that did not change with age. Across the three age groups, a more robust right-hand preference was observed for the unimanual, grasp-to-eat task. To disentangle if the nature (unimanual) or the end-goal (grasp-to-eat) was the driver of the robust right-hand preference, a follow up experiment was conducted. Young-adult participants completed a unimanual grasp-to-place task. This was contrasted with the unimanual grasp-to-eat task and the bimanual grasp-to-construct task. Rates of hand preference for the grasp-to-eat task remained the highest when compared to the other two grasping tasks. Together, the results demonstrate that hand preference remains stable from childhood to older adulthood, and they suggest that a left hemisphere specialization exists for grasping, particularly when bringing food to the mouth.

## Introduction

Research on human handedness has revealed a preference to use the right hand. These investigations have used a variety of methods, such as the Annett Peg Moving Task (Annett et al., 1979), the Block Building Task (Gonzalez and Goodale, 2009; Stone et al., 2013; Stone and Gonzalez, 2014), the Task Complexity Gradient (Gooderham and Bryden, 2014), and the Tapley–Bryden Dot Marking Task (Tapley and Bryden, 1985), as well as numerous paper-based questionnaires (Oldfield, 1971; Steenhuis and Bryden, 1989; Brown et al., 2006). This right-hand preference for grasping is sensitive to multiple factors, including the nature of the task (i.e., unimanual or bimanual), the end-goal of the task (e.g., grasp to throw, place, or use the object), and the space in which the target object is located [i.e., ipsilateral (on the same side) or contralateral (on the opposite side)] with respect to the grasping hand.

Right-hand use has been shown to be more pronounced for unimanual tasks in which participants are required to pick up one object at a time (Bishop et al., 1996; Corballis, 1997; Calvert and Bishop, 1998; Gabbard et al., 2003; Bryden and Roy, 2006; Carlier et al., 2006; Sacrey et al., 2012) than during tasks in which both hands could potentially be engaged (Gonzalez and Goodale, 2009; Stone et al., 2013; Stone and Gonzalez, 2014). Furthermore, the intent behind an action (i.e., the end-goal of a grasping action; what the individual plans to do with the object after it has been grasped) can also affect hand preference (Geerts et al., 2003; Mamolo et al., 2004, 2006; Bryden and Roy, 2006; Rat-Fischer et al., 2013; Sacrey et al., 2013). For example, Mamolo et al. (2006) found that when right-handed individuals reached for a tool with the intent to use it (compared to just picking it up), right-hand preference increased significantly. In contrast, when individuals were asked to grasp various toys with either the intent to throw it outwards or place it in a nearby box, hand use did not differ between the conditions Bryden and Roy (2006). Similar results have been reported in children. In a recent study, for example, children 1–5 years old were asked to reach for, grasp, and eat cereal (Cheerios® and Froot Loops®), or reach for and grasp blocks in order to manipulate them and build a structure. A right-hand preference was observed in one-year-old children, but only for the grasp-to-eat task. This preference did not surface for the grasp-to-construct task until 4 years of age, at which time it was suggested that it resembled adult behavior (Sacrey et al., 2013).

In addition to the nature and end-goal of a grasp, an object's location in space has also been shown to play an important role in hand selection. For biomechanical reasons, it would make more sense for one to grasp an object with the hand ipsilateral to the object (i.e., right hand for objects in the right space and the left hand for objects in left space). Contrary to this speculation, many researchers have shown that right-hand contralateral grasps in left space are quite common (Leconte and Fagard, 2004; Bryden and Roy, 2006; Mamolo et al., 2006; Gonzalez et al., 2007; Bryden and Huszczynski, 2011; Stone et al., 2013) which refutes the biomechanical speculation. Considering all these factors (task nature, end-goal, and space use), mixed conclusions have been drawn regarding hand preference, and handedness has been thus referred to as a “multifaceted biosocial developmental process” (Michel et al., 2013). Perhaps a way to understand the complexity of hand use/preference is to document its developmental trajectory and examine how the nature and end-goal of the task as well as object location may influence this preference over time.

Given that the vast majority of studies investigating hand dominance have been on developing children or young adults (Annett, 1970; Briggs and Nebes, 1975; Michel, 1981; Fagard and Marks, 2000; Cavill and Bryden, 2003; Bryden and Roy, 2006; Hill and Khanem, 2009; Jacquet et al., 2012; Sacrey et al., 2013; Stone et al., 2013; Scharoun and Bryden, 2014; Stone and Gonzalez, 2014), less is known about changes in hand preference into older adulthood (55+ years) particularly when using objective measures. Most studies, to our knowledge, have used subjective measures (i.e., questionnaires or interviews) to document changes in hand preference in older adults. These studies have reported that with age there is an increase in (the perception of) dominant hand use in right-handers and a decrease in left-handers (Porac et al., 1980; Beukelaar and Kroonenberg, 1986; Hugdahl et al., 1993, 1996; Porac, 1993; Coren, 1995; Porac and Friesen, 2000; Porac and Searleman, 2002, 2006; Hatta et al., 2005; Kumar et al., 2010). The few studies that have objectively tested hand preference in older adults have presented seemingly conflicting results. One reported that the tendency to prefer one hand over the other increases with age (Weller and Latimer-Sayer, 1985), while another concluded that hand-preference lateralization actually decreases as one ages (Kalisch et al., 2006). Furthermore, a recent investigation showed no change in hand preference with age (Gooderham and Bryden, 2014). These studies however, only measured hand preference for unimanual tasks. So, not only are there conflicting results from the few studies that have objectively investigated hand preference across different ages, but we have yet to form a clear picture on whether and how this preference may change as a function of task demands (e.g., nature, end-goal, and space). The main goal of the current investigation was to address this gap in knowledge.

To determine how task nature, end-goal, and object location influence hand preference for grasping across the lifespan, participants aged 2–90 were tested on a unimanual and a bimanual task while hand preference was recorded. For the unimanual task we chose a grasp-to-eat action and for the bimanual task a grasp-to-construct action. Grasp-to-eat was chosen because previous research has shown an earlier emergence of right-hand preference for this action when compared to grasp-to-construct (Sacrey et al., 2013). In the current investigation we used methodology similar to that used in previous reports (Gonzalez et al., 2007; Gonzalez and Goodale, 2009; Sacrey et al., 2013; Stone et al., 2013; Stone and Gonzalez, 2014). For the grasp-to-eat task, participants picked up Froot Loops® unimanually from a tabletop in order to eat them. For the grasp-to-construct task participants were required to pick up building blocks (LEGO®) from a tabletop in order to construct a simple 3D block model. In our previous investigations (see Stone et al., 2013; Stone and Gonzalez, 2014) we have characterized the grasp-to-construct task as *bimanual asymmetric* because the interaction of the two hands is necessary in order to complete it efficiently. Typically one hand is used for grasping the blocks while the other stabilizes the model under construction. Using these two tasks allowed us not only to address the question of whether hand preference changes with age, but also to assess how hand preference is influenced by (1) end-goal (eat vs. construct); (2) task nature (unimanual vs. bimanual); and (3) space use (ipsilateral and contralateral space).

## Experiment one

### Methods and procedures

#### Participants

A total of 142 right-handed (by self or parent report) individuals were included in the study and placed into one of three age groups: *Children:* (*n* = 80) ranging from 2 to 11 (50 female), *Young-Adults:* (*n* = 37) ranging from 17 to 28 (25 female), *Older-Adults:* (*n* = 25) ranging from 57 to 90 (16 female) years of age. Children and older adults were recruited from the community of Lethbridge and young-adults from the University of Lethbridge. The study was approved by the University of Lethbridge Human Subjects Research Committee (protocols #2013-040, #2011-022, and #2012-006) and all participants or caregivers gave written informed consent in accordance with the Declaration of Helsinki. Participants were naïve to the purposes of the study.

#### Apparatus and stimuli

***Handedness questionnaire***. A modified version (Stone et al., 2013) of the Edinburgh (Oldfield, 1971) and Waterloo (Brown et al., 2006) handedness questionnaires was given to all participants or caregivers at the end of the experiment. Items in the questionnaire were rated on a scale [+2 (right always) +1 (right usually), 0 (equal), −1 (left usually) and −2 (left always)] depending on how much a hand was preferred for a particular task. Each response was scored as 2, 1, −1, or −2 and a total score was obtained by adding all values. This version included questions on hand preference for up to 22 (ranging from 11 to 22) different tasks. Young and older adults received the questionnaire with 22 items. Children received a questionnaire containing 11 (2–4 years old) or 17 questions (5–11 years old) as not all questions in the questionnarie were appropriate for young children. All scores are expressed as percentage of the highest possible score from the total number of questions answered.

**Figure 1:**
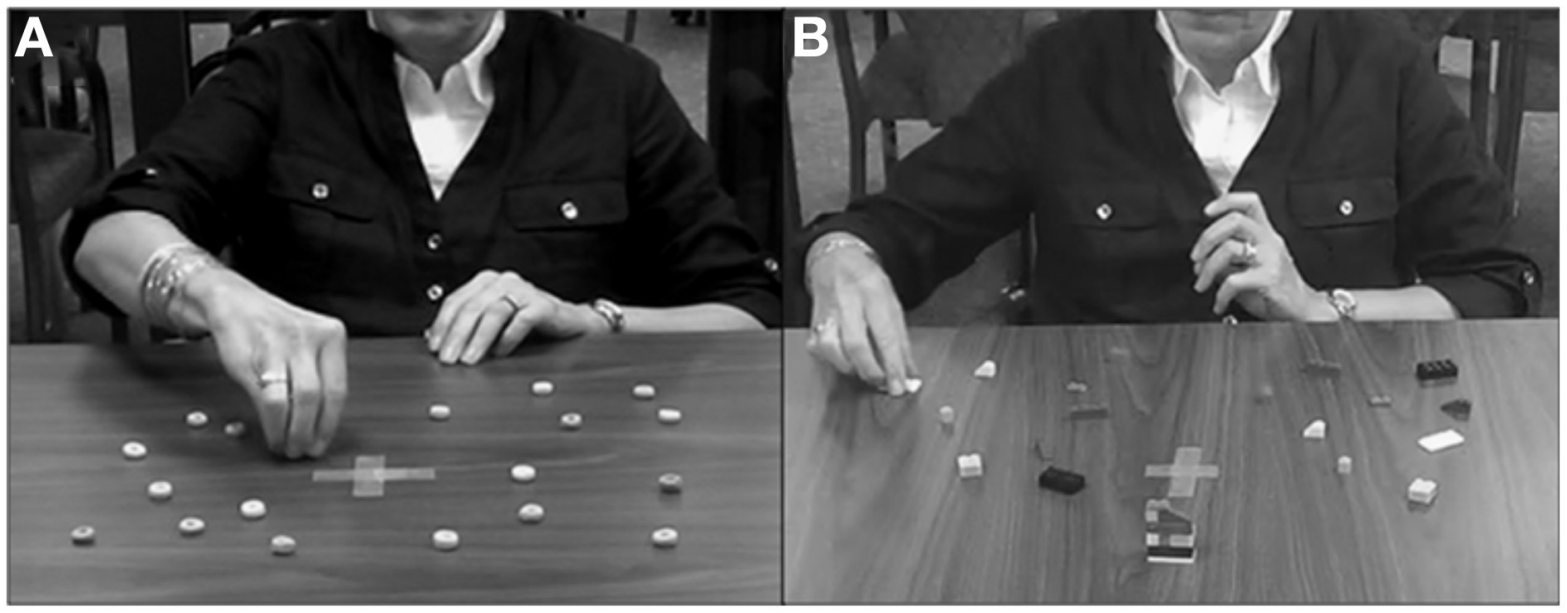
**Experimental set up for the (A) Grasp-to-eat and (B) Grasp-to-construct tasks**.

***Grasp-to-eat***. A total of 20 Froot Loops® were used for the experiment (except for the 2-year-olds who consumed 10 loops). Five of six different colors of loops were placed on the table: purple, pink, orange, yellow, blue, or green. The loops were distributed evenly onto the left and right sides of the table (10 loops per side, 2 of each color; see Figure 1A and Supplementary Videos 1, 2). The table was disinfected prior to the task for each participant. The experimenter wore a pair of gloves when placing the loops on the tabletop.

***Grasp-to-construct***. A total of three models were used for the experiment. Each model contained 5–10 blocks of various colors and shapes. Children aged 2–3 were presented with Mega Bloks® (3.1-6.3 L × 3.1 W × 2.0 cm H) whereas 4 years of age and up were presented with Lego® blocks (ranging in size from < 1.5 L × 0.7 W × 1.0 cm H to 3.1 L × 1.5 W × 1.0 cm H). A previous study in 3- to 5-year-old children showed no difference in hand use between the two different types of blocks (Sacrey et al., 2013). All blocks that made up the models were scattered on a table with a working space of 70 cm deep × 122 cm wide for the adults, and 60 cm deep × 80 cm wide for the children. The blocks were distributed evenly onto the left and right sides of the table (see Figure 1B). No blocks were re-organized between models or replaced after their use (see Supplementary Videos 3–8 for examples of this task).

#### Procedures

***Grasp-to-eat***. Participants were seated in front of the table facing the middle of the display. They were then instructed to pick up and eat one loop of a specific color (e.g., a pink loop). Given this instruction participants used only one hand at a time. It was at the participant's discretion to chose with which hand to grasp. This instruction was repeated until no loops remained on the tabletop (see Supplementary Videos 1, 2). No other instruction was given. Therefore, the participant grasped for and consumed 20 loops (i.e., four loops of each color or two loops of each color for the 2-year-olds). The order of colored loops to be grasped was randomized between participants.

***Grasp-to-construct***. Participants were seated in front of the table facing the middle of the display. A model was placed centrally, approximately arm's length away from the participant. Next, participants were instructed to replicate the model as quickly and accurately as possible from the blocks given on the table. No other instruction was given. Once the model was replicated, both the original and the constructed models were removed from the table and a new model was presented. 2- and 3-year olds who were unable to accurately make a replica of the given model built a model of their choice until all the blocks were used. Used blocks were not replaced after each model was completed. Each participant built three models in total. Model presentation was counterbalanced among participants.

***Data Analysis***. Both tasks (grasp-to-eat and grasp-to-construct) were recorded on a JVC HD Everio video recorder approximately 160 cm away from the individual with a clear view of the tabletop, target objects (blocks or loops), and participants' hands. All recorded videos were analyzed offline. Each grasp was recorded as a left- or right-hand grasp in the participants' ipsilateral or contralateral space. The total number of grasps was counted to determine a percent for right-hand use (number of right grasps/total number of grasps × 100). Data were analyzed using SPSS Statistics 19.0 for Windows (SPSS Inc., Chicago, IL, USA). Mean and standard errors are reported in percentage for *all* analyses. Bonferroni correction was applied to comparisons where applicable.

### Results

No effect of sex was found in any of the analyses, therefore female and male data were combined.

#### Handedness questionnaires

A One-Way analysis of variance (ANOVA) with Group (Children, Young-Adults, and Older-Adults) as the independent variable and scores from the handedness questionnaire as the dependent variable was conducted. Results show a main effect of Group [*F*_(2, 141)_ = 7.54; *p* = 0.001]. *Post-hoc* analyses revealed that Older-Adults scored higher in the questionnaire (91.6 ± 1.6) than Younger-Adults (71.3 ± 2.3; *p* = 0.008) and Children (77.18 ± 2.8; *p* = 0.001). Scores in the handedness questionnaire did not differ between Children and Younger-Adults (*p* = 0.45).

#### Grasp-to-eat

***Overall right-hand use***. Children showed no difficulties in discriminating the loops by color. A One-Way ANOVA with Group (Children, Young-Adults, and Older-Adults) as the independent variable and right-hand use as the dependent variable revealed no significant effect of group [*F*_(2, 141)_ = 0.3; *p* = 0.72]. In other words, Children, Young-Adults, and Older-Adults displayed similar rates of right-hand use when picking up the loops to eat (81.3 ± 2.4; 84.1 ± 3.3; 84.3 ± 4.3, respectively).

***Contralateral grasps***. To assess if hand use changes as a function of space (ipsilateral/contralateral) a repeated measures ANOVA with Group (Children, Young-Adults, and Older-Adults) as the between factor and Hand (Right and Left) used to grasp in contralateral space as the within factor was performed. Results revealed a significant main effect of Hand [*F*_(1, 139)_ = 250.7; *p* < 0.0001], no main effect of group [*F*_(2, 139)_ = 0.1; *p* = 0.8] and no significant interaction [*F*_(2, 139)_ = 0.2; *p* = 0.8]. The right hand was used much more to cross the midline and grasp the loops placed on the left side of the table than the left hand was to grasp the loops on the right side of the table (34.9 ± 1.6 vs. 2.3 ± 0.6). Overall contralateral grasp percentage was similar across all three age groups (Children: 18.3 ± 0.8; Young-Adults: 18.3 ± 1.3; Older-Adults: 19.3 ± 1.6).

#### Grasp-to-construct

***Overall right-hand use***. A One-Way ANOVA with Group (Children, Young-Adults, and Older-Adults) as the independent variable and right-hand use as the dependent variable, revealed no significant effect of group [*F*_(2, 141)_ = 2.1; *p* = 0.11]. In other words, Children, Young-Adults, and Older-Adults displayed similar rates of right-hand use when picking up the blocks (67.2 ± 1.7; 64.7 ± 1.6; 72.7 ± 3.6, respectively).

**Figure 2:**
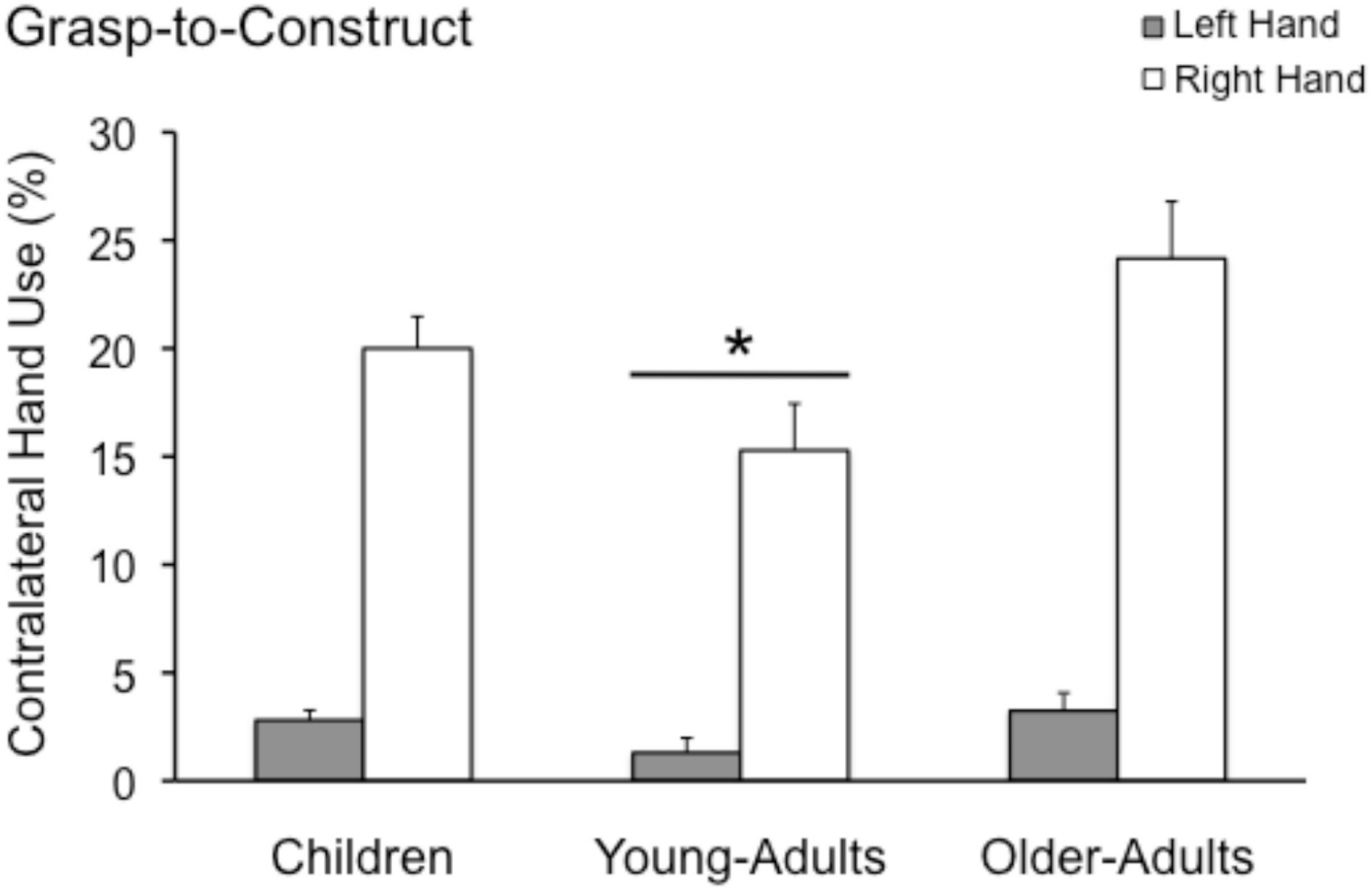
**Mean and standard error (in %) for left and right contralateral grasps observed in the grasp-to-construct task by children, young-adults, and older-adults**. Note that the young-adults made significantly fewer contralateral grasps when compared to the other two groups. ^*^p < 0.01.

***Contralateral grasps***. A repeated measures ANOVA with Group (Children, Young-Adults, and Older-Adults) as the between factor and Hand (Right and Left) used to grasp in contralateral space as the within factor was performed. Results (see Figure 2) revealed a significant main effect of Hand [*F*_(1, 139)_ = 152.2; *p* < 0.0001], a main effect of group [*F*_(2, 139)_ = 5.9; *p* = 0.003] but no significant interaction [*F*_(2, 139)_ = 1.6; *p* = 0.2]. The right hand was used much more to cross the midline and grasp the blocks placed on the left side of the table than the left hand was to grasp the blocks on the right side of the table (19.8 ± 1.2 vs. 2.4 ± 0.3). Pairwise comparisons revealed that the Young-Adult group (8.2 ± 1.0) crossed the midline less often than the Children (11.4 ± 0.7; *p* = 0.04) and the Older-Adult (13.7 ± 1.2; *p* = 0.003) groups. Children and Older-Adults did not differ from each other (*p* = 0.33).

#### Grasp-to-eat vs. grasp-to-construct comparisons

**Figure 3:**
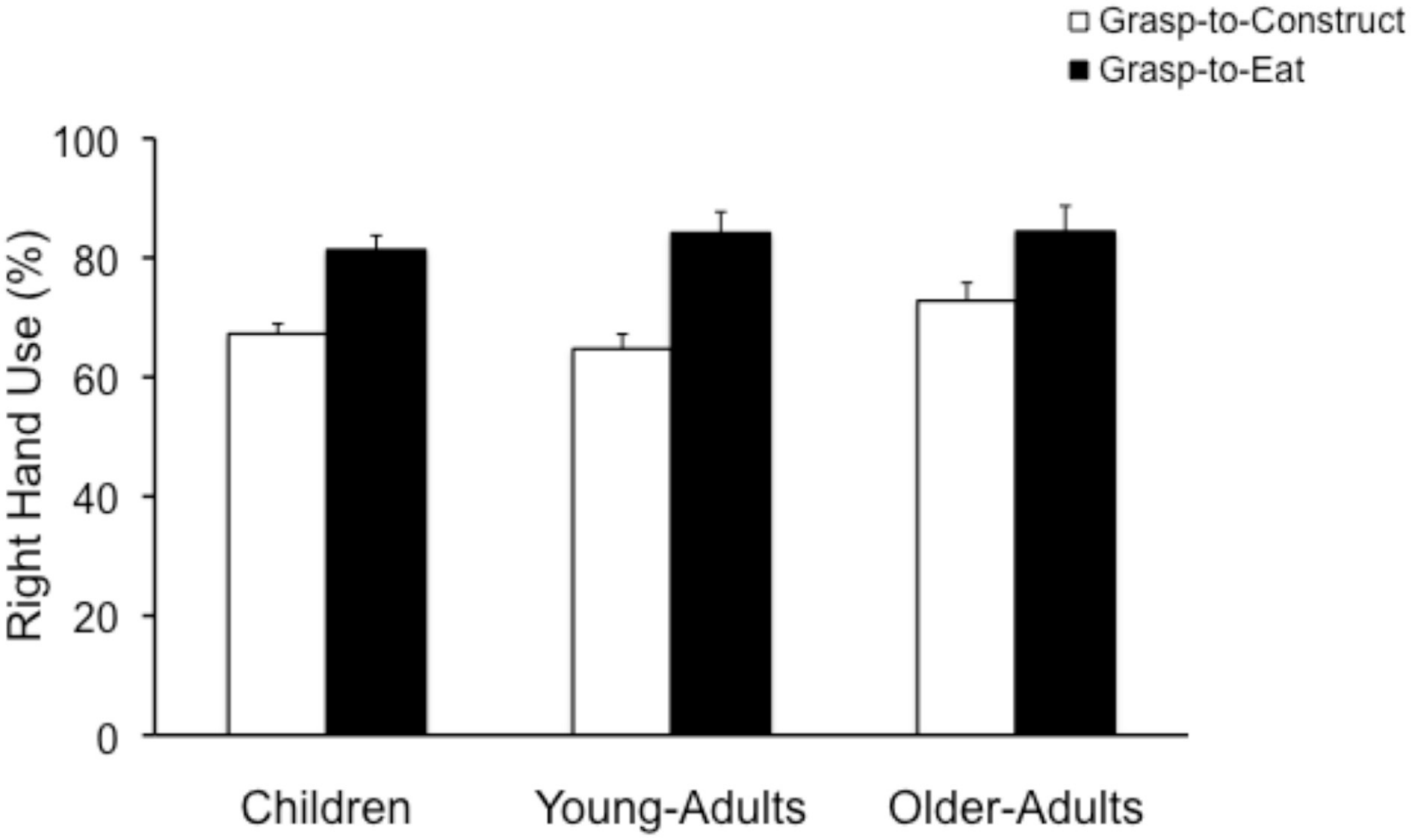
**Mean and standard error (in %) for right-hand use in the grasp-to-construct and the grasp-to-eat tasks by children, young-adults, and older-adults**. Note the increase in right-hand use for the grasp-to-eat task across the three groups.

***Overall right-hand use***. A repeated measures ANOVA with end-goal (grasp-to-eat and grasp-to-construct) as the within factor and Group (Children, Young-Adults, and Older-Adults) as the between factor, revealed a significant main effect of end-goal [*F*_(1, 139)_ = 47.62; *p* < 0.0001], no main effect of group [*F*_(2, 139)_ = 0.88; *p* = 0.41] and no significant interaction [*F*_(2, 139)_ = 1.0; *p* = 0.36]. Right-hand use was greater for the grasp-to-eat task (83.2 ± 2.0) when compared to the grasp-to-construct task (68.2 ± 1.4; see Figure 3).

***Contralateral grasps***. A repeated measures ANOVA with end-goal (grasp-to-eat and grasp-to-construct) and hand (Right, Left) used to grasp in contralateral space as the within factors and group (Children, Young-Adults, and Older-Adults) as the between factor was conducted. Similar to the overall right-hand use, there was a main effect of end-goal [*F*_(1, 139)_ = 77.4; *p* < 0.0001], no main effect of group [*F*_(2, 139)_ = 2.5; *p* = 0.08], and a significant effect of hand [*F*_(1, 139)_ = 321.2; *p* < 0.0001]. The only significant interaction was the end-goal by hand [*F*_(1, 139)_ = 49.9; *p* < 0.0001] wherein the right hand was used more often to cross the midline in the grasp-to-eat task (18.6 ± 0.7) when compared to the grasp-to-construct task (11.1 ± 0.5).

#### Correlations

To investigate the possible relationship among age (chronological age 2–90), hand use for the two grasping tasks, and scores on the handedness questionnaire, a correlation (Pearson's *r*) was conducted on these variables. Table 1 shows a positive correlation between chronological age and questionnaire scores (*r* = 0.29; *p* < 0.0001). The older the individual, the more they reported to use their right hand on the items in the questionnaire. The correlation between chronological age and right-hand use in the grasp-to-construct task approached significance (*r* = 0.16; *p* = 0.056). The older the age, the more the right hand was used for picking up the blocks. Not surprisingly, the correlation between the two grasping tasks was significant (*r* = 0.23; *p* = 0.005): the more the right hand was used to pick up blocks, the more it was used to grasp loops.

**Table 1:** **Correlation matrix of all variables**.

	**Age**	**Lego**	**Eat**	**Handedness questionnaire**
Age	1	0.161^♦^	0.076	0.291^**^
Grasp-to-construct		1	0.235^**^	0.048
Grasp-to-eat			1	0.052
Handedness questionnaire				1

♦ *Approaching significance at value of 0.056*.

** *Correlation is significant at the 0.01 level (2-tailed)*.

Because hand preference has been shown to change during childhood (Coren et al., 1981; McManus et al., 1988; Gooderham and Bryden, 2014) correlation analyses between age and right-hand use in the grasp-to-construct and grasp-to eat tasks were performed on each age group (children, young-adults, and older-adults). The results showed that correlations between age and right-hand use in the grasp-to-eat and grasp-to-construct tasks approached significance *only* in the children group (*r* = 0.211; *p* = 0.06; *r* = 0.219; *p* = 0.051). There were no significant correlations between age and right-hand use in either grasping task in the young- and older-adult groups (all *p* > 0.15). We followed-up the near significant correlation in children by sub-dividing this group again by age: 2–4, 5–8, and 9–11 years of age. A One-Way ANOVA showed no significant difference between the groups in hand preference for either grasping task [grasp-to-eat: *F*_(2, 79)_ = 0.7; *p* = 0.4; grasp-to-construct: *F*_(2, 79)_ = 0.8; *p* = 0.4]. So although the correlations approached significance, the group analysis was far from significant. We speculate that this is due to the high variability (with standard deviations ranging from 14 to 24%; see Table 2) within each sub-group. Importantly and highlighting the main finding of the current study, the between-task differences (grasp-to-eat vs. grasp-to-construct) were significant in all sub-groups of children (*p* < 0.01).

**Table 2:** **Percent of total grasps completed with the right hand in each task by children aged 2–11**.

**Sub-group (age in years)**	***N***	**Grasp-to-construct (%)**	**Grasp-to-eat (%)**
2–4	21	64.2 ± 3.2	78.0 ± 4.5
5–8	31	66.5 ± 2.9	80.0 ± 4.3
9–11	28	70.2 ± 2.9	81.3 ± 2.4

*Values reported are means ± standard errors of each age group. Note that there were no significant differences between sub-groups, but there were significant differences between the two grasping tasks within each sub-group*.

### Discussion

**Figure 4:**
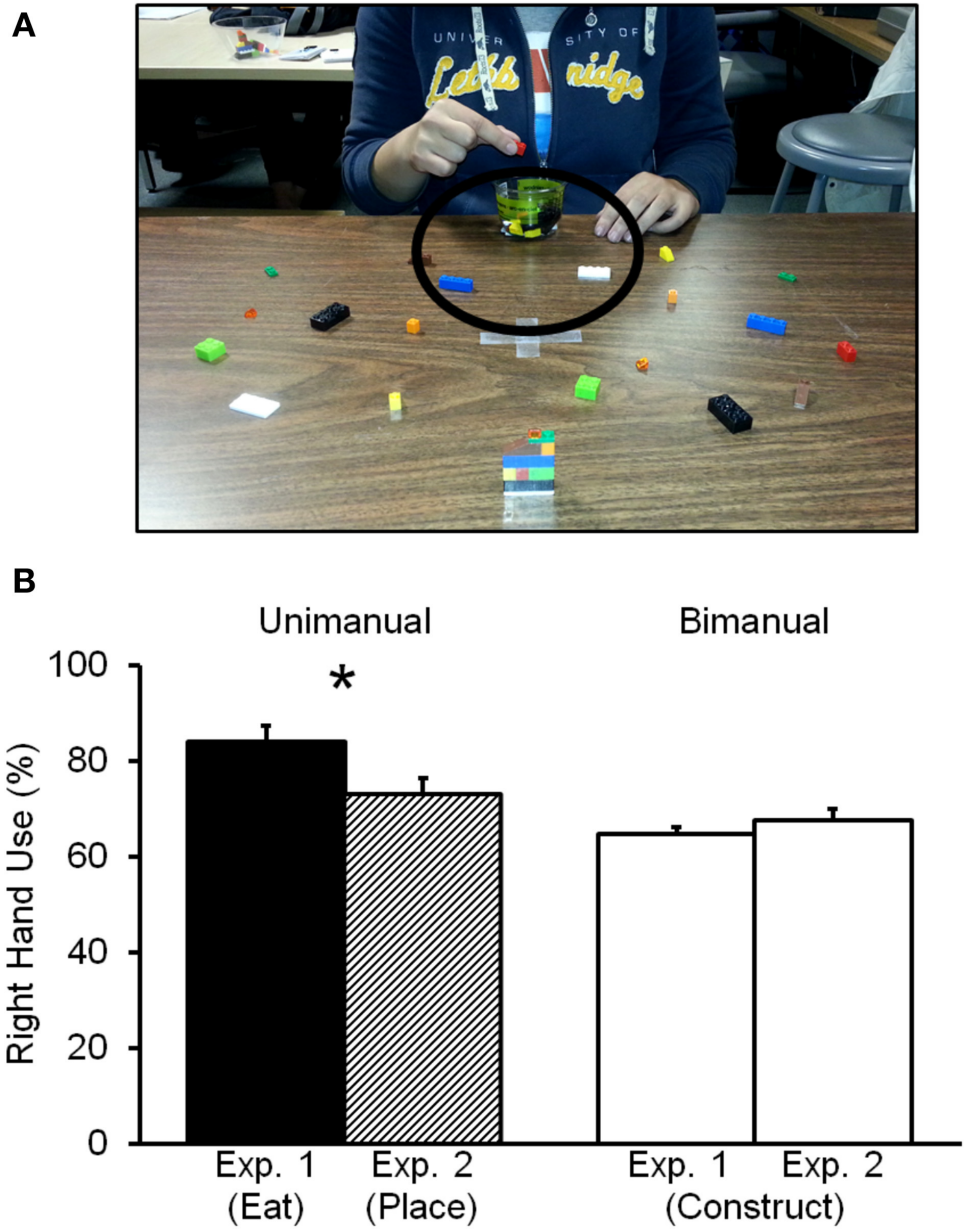
**(A)** Experimental set up for the Grasp-to-place task (Experiment Two). **(B)** Mean and standard error (in %) for right-hand use in the grasp-to-construct and the grasp-to-eat tasks (Experiment One) and the grasp-to-place task (Experiment Two). ^*^p = 0.02.

The results demonstrated clear differences in right-hand use between the grasp-to-eat and grasp-to-construct actions. Participants in the three age groups displayed greater right-hand preference when picking up the object with intent to eat (loops) vs. picking up the object with intent to construct. This result aligns with a previous report in children that showed increased rates of right-hand use for grasping food vs. blocks (Sacrey et al., 2013). The result from the present study also reinforces the idea that grasp-to-eat actions might be at the origin of population level right-handedness (Flindall and Gonzalez, 2013; Flindall et al., 2014). These investigations of hand kinematics have shown evidence that the grasp-to-eat action executed with the right—but not the left-hand elicits smaller grip apertures during the hand pre-shaping phase of the grasp when compared to other grasping movements. Because smaller grip apertures are typically associated with greater precision, this finding was interpreted as a right-hand advantage for the grasp-to-eat movement. Given this interpretation, it is feasible to speculate that in the current study the greater use of the right hand for the grasp-to-eat task could be related to this kinematic advantage. With respect to our original question however, the results do not resolve if this increase in right-hand use is exclusively due to the end-goal of bringing the object to the mouth. One could argue that the grasp-to-eat task only requires one hand to complete whereas the grasp-to-construct task requires the interaction of both hands. It is therefore possible that the increase in right-hand use is simply due to the unimanual nature of the grasp-to-eat task. To address this possibility, young adult participants were asked to complete the grasp-to-construct task (identical to that in Experiment One) and we contrasted their hand preference to a unimanual version of the same task. For the latter task, the same models were presented to participants, but instead of constructing, they were asked to pick up each block (one at a time) that composed the model and place the block into a container near their body (Figure 4). If the unimanual nature of the grasp-to-eat task is what drives the robust right-hand preference, then one would expect similar rates of right-hand use when participants are bringing the block to the container. But, if instead, right-hand use in this unimanual action is lower when compared to the grasp-to-eat task then it would suggest that bringing food to the mouth is lateralized to the right hand.

## Experiment two

### Methods and procedures

#### Participants

Because Experiment One showed no difference in hand preference for grasping among the three age groups (Children, Young-Adults, Older-Adults), only Young-Adults were tested in the second experiment. A total of 37 self-reported right-handed individuals from the University of Lethbridge were included in this study. Participants ranged in age from 17 to 34 (mean age: 20.9 ± 0.5 years). The study was approved by the University of Lethbridge Human Subjects Research Committee (protocol #2011-022) and all participants gave written informed consent in accordance with the Declaration of Helsinki. Participants were naïve to the purposes of the study.

#### Apparatus and stimuli

***Handedness questionnaire***. The questionnaire was the same as in Experiment One.

***Grasp-to-construct***. This task was the same as in Experiment One.

***Grasp-to-place***. This task was set up the same as the grasp-to-construct task, with one modification: a short, clear cup was placed in the front and center of the participant.

#### Procedures

***Grasp-to-construct***. Procedures for this task were identical to those in Experiment One.

***Grasp-to-place***. Participants were seated in front of the table facing the middle of the display. A model was presented centrally, approximately arm's length away from the participant (as in Experiment One). Next, participants were instructed to pick up each block (one at a time) that made up the presented model and place it into the container as quickly as possible. Once all the blocks that made up the model were inside the container, the model was removed and a new model was presented (see Supplementary Video 9). No blocks were replaced after each model was completed. Each participant picked up 30 blocks (10 blocks per model). Model presentation was counterbalanced among participants. As in Experiment One, both tasks were recorded on a JVC HD Everio video recorder approximately 160 cm away from the individual with a clear view of the tabletop, target objects, and participants' hands.

### Results

No effect of sex was found in any of the analyses, therefore female and male data were combined.

#### Handedness questionnaires

The mean score in the questionnaire was 73.3 ± 2.2. To investigate if this group was different from the Young-Adults in Experiment One, a One-Way ANOVA with Experiment (One, Two) as the independent variable and scores from the handedness questionnaire as the dependent variable was conducted. Results show no difference between the two groups [*F*_(1, 73)_ = 0.3; *p* = 0.5].

#### Grasp-to-construct (bimanual) vs. grasp-to-place (unimanual) comparisons

A paired samples *t*-test revealed no significant difference between the two tasks [*t*_(36)_ = −1.6; *p* = 0.1; 67.6 ± 2.5 and 73.0 ± 3.5, respectively]. In other words participants used their right hands to the same extent in both tasks even though one task was strictly unimanual.

#### Comparison between Experiment One and Experiment Two

To investigate if the finding of greater right-hand use in the grasp-to-eat task (Experiment One) was due to the end-goal of the task (bringing it to the mouth) OR to the intrinsic unimanual nature of the task, we conducted a repeated measures ANOVA. Experiment One and Experiment Two served as the between factors and Task Nature (unimanual, bimanual) as the within factors. This analysis allowed us to investigate if the end-goal of the action influences hand use given that in Experiment One the end-goal was to bring the item to the mouth and in Experiment Two the end-goal was to bring the object to a container. Crucially, in both cases the task was unimanual in nature. A main effect of Task Nature was found [*F*_(1, 72)_ = 26.7; *p* < 0.0001], indicating that participants used the right hand more often during the unimanual task regardless of the Experiment (unimanual: 78.6 ± 2.4; bimanual: 66.1 ± 1.5). There was no main effect of Experiment [*F*_(1, 72)_ = 1.4; *p* = 0.2], but a significant interaction [*F*_(1, 72)_ = 8.4; *p* = 0.005; Figure 4]. To investigate this interaction, a series of *post-hoc* analyses (paired-samples- and independent-*t*-tests) were conducted. First, an independent *t*-test showed no difference in right-hand use between the bimanual (grasp-to-construct) tasks of Experiment One and Two [*t*_(72)_ = −0.9; *p* = 0.3]. Importantly, the analysis for the unimanual tasks (grasp-to-eat vs. grasp-to-place) between Experiment One and Two *was* significant [*t*_(72)_ = 2.2; *p* = 0.02]. This result suggests that the end-goal and NOT the unimanual nature of these tasks determined the rate at which the right hand was used, namely greater for the grasp-to-eat action. Moreover, and in contrast to Experiment One, paired-samples *t*-tests revealed that there was no difference between the unimanual (grasp-to-place) and the bimanual task (grasp-to-construct) in Experiment Two [*t*_(36)_ = −1.6; *p* = 0.1]. This result further supports the idea that the grasp-to-eat action might be at the origin of population-level right-handedness.

## General discussion

To investigate how hand preference for grasping changes as a function of task throughout the lifespan, right-handed participants in three age groups (children, aged 2–11; young adults, aged 18–21; and older adults, aged 57–90) were recorded while performing unimanual self-feeding and bimanual construction tasks. In the unimanual self-feeding task, participants were required to grasp-to-eat Froot Loops®, one at a time, from a pseudo-symmetrical array before them. In the bimanual construction task, participants were required to grasp building blocks from a pseudo-symmetrical array to replicate simple models. Hand preference for these tasks was recorded and analyzed offline to determine the influence of age, task nature (unimanual vs. bimanual), end-goal (build vs. eat), and object location (ipsilateral or contralateral to the grasping hand) on the regulation of hand use. The results showed two interesting findings: first, no difference in hand preference among groups, demonstrating a stable right-hand preference in children, young- and older-adults. Second, right-hand preference was greater for the unimanual grasp-to-eat task when compared to the bimanual grasp-to-construct task. To investigate whether this increased preference was due to the unimanual nature of the task or the end-goal of the action, a secondary experiment was conducted wherein participants performed a unimanual grasp-to-place task to compare to the unimanual grasp-to-eat action. Right-hand preference in the unimanual grasp-to-place task was similar to that of the bimanual grasp-to-build task, both lower than the right-hand preference for the grasp-to-eat task. Finally, analysis of contralateral grasps revealed that participants performed contralateral grasps with their right hands more often than they did with their left hands, although young adults performed significantly fewer contralateral grasps than both children and older adults. Taken together, the results show that hand preference, is significantly affected by the end-goal and spatial demands of the task, but does not change over the course of one's lifespan. Furthermore, they suggest that compared to other seemingly similar movements, the grasp-to-eat action is more lateralized to the right hand.

Few studies have examined changes in hand use across the lifespan. In an early study on age-related changes to manual dexterity, Weller and Latimer-Sayer (1985) found that right-hand motor skills were better preserved than left-hand motor skills in participants of advanced age. When combined with an overall decrease in manual dexterity, this study suggests that the gap between right- and left-hand performance increases as one ages. In contrast, a more recent study by Kalisch et al. (2006) found that dominant-hand speed and precision advantages observed in young adults are lost later in life, and that this change is accompanied by a shift in hand-preference for commonplace tasks. In other words, that study found that as right-hand advantage declines, so does right-hand preference in favor of a more ambidextrous approach to everyday activities. A third study, by Gooderham and Bryden (2014) used a multifaceted approach to measuring hand dominance in a large cross-sectional study and found that, once firmly established in adulthood, the level and degree of hand-dominance does not change with increasing age. The results of the current study support that finding, as hand-preference for the grasp-to-eat, grasp-to-construct, and grasp-to-place tasks showed no age-related changes between any of our groups. In contrast with Gooderham and Bryden (2014), however, we observed no difference in laterality between children and other age groups in the behavioral task. This may be due in part to the differences in ages between children in both studies; Gooderham and Bryden tested children aged 2–4, whereas the age of our sample of children ranged from 2 to 11 years old. The 11-year-old children would have been considered young adolescents by Gooderham and Bryden. More likely, the difference in results may stem from methodological differences between the studies. Gooderham and Bryden inferred hand-dominance through motor-skill performance and a complexity-related switch point, whereas we asked participants to perform simple everyday activities and inferred lateralization of dominance from direct observation of hand preference. With regards to the handedness questionnaire, the significant correlation between age and handedness scores suggest that the older the individual the more right-handed they perceive themselves to be. This is in agreement with a previous report which concluded that elderly subjects rate themselves as strongly right-handed regardless of their objective hand use (Kalisch et al., 2006). The primary finding of our study is consistent with these results, in that lateralization of hand dominance neither increases nor decreases as one ages beyond adulthood, regardless of one's subjective perception.

The second finding of the current study was that the end-goal of an action plays a significant role in determining whether or not one will use their right hand to perform that action. In the unimanual self-feeding task, participants of all ages used their right hands significantly more often than they did during the bimanual construction or unimanual placement tasks. This increased right-hand preference also extended to contralateral grasps, which were significantly more common when the objective of the grasp was to eat, rather than place or manipulate the target. As the mechanical requirements of the different types of grasp are ostensibly identical, the decision to more often use the right-hand for grasp-to-eat tasks suggests a fundamental difference between the neurological origins of the grasps. This presumption is supported by findings from Sacrey et al. (2013), who found that children develop a right-hand preference for grasp-to-eat tasks several years earlier than they do for grasp-to-build tasks. Furthermore, Flindall and Gonzalez (Flindall and Gonzalez, 2013, 2014, submitted; Flindall et al., 2014) have found a left-hemisphere/right-hand advantage in the kinematics of grasp-to-eat/hand-to-mouth actions that is absent from grasp-to-place actions. Specifically, when grasping a small food item with intent to eat, participants produce tighter maximum grip apertures during the outgoing movement than when grasping the same item to place it in a receptacle near the mouth. This task difference in hand pre-shaping is predominantly lateralized to the right hand, regardless of a person's overall hand preference (Flindall and Gonzalez, 2013; Flindall et al., 2014). Taken together, these findings all support a theory of human motor cortex organized around a catalog of movements based on end-goal, rather than mechanical requirements (Graziano et al., 2002, 2004, 2005; Fogassi et al., 2005; Graziano, 2006, 2009; Bonini et al., 2011, 2012; Flindall and Gonzalez, 2013, 2014). The results from the present study demonstrating greater right-hand use for the grasp-to-eat task further support the proposal that this type of action might be at the forefront of population level right-handedness in humans.

In conclusion, the current study investigated lateralization of motor dominance as it relates to task nature, end-goal, and space constraints by observing hand preference in simple grasp-to-eat, grasp-to-construct, and grasp-to-place tasks. To assess whether and how hand preference changes throughout the lifespan, these tasks were performed by children, young adults, and seniors. A right-hand preference for all tasks was observed, however, this preference was greater during the grasp-to-eat task. This effect was consistent throughout all age groups. These results further our knowledge of the developmental trajectory of manual asymmetries across the lifespan.

### Conflict of interest statement

The authors declare that the research was conducted in the absence of any commercial or financial relationships that could be construed as a potential conflict of interest.

## References

[B1] Annett J. Annett M. Hudson P. T. W. Turner A. (1979). The control of movement in the preferred and non-preferred hands. Q. J. Exp. Psychol. 31, 641–652. 10.1080/14640747908400755 534286

[B2] Annett M. (1970). A classification of hand preference by association analysis. Br. J. Psychol. 61, 303–321. 10.1111/j.2044-8295.1970.tb01248.x 5457503

[B3] Beukelaar L. J. Kroonenberg P. M. (1986). Changes over time in the relationship between hand preference and writing hand among left-handers. Neuropsychologia 24, 301–303. 10.1016/0028-3932(86)90066-7 3714038

[B4] Bishop D. V. Ross V. A. Daniels M. S. Bright P. (1996). The measurement of hand preference: a validation study comparing three groups of right-handers. Br. J. Psychol. 87 (Pt 2), 269–285. 10.1111/j.2044-8295.1996.tb02590.x 8673359

[B5] Bonini L. Serventi F. U. Bruni S. Maranesi M. Bimbi M. Simone L.. (2012). Selectivity for grip type and action goal in macaque inferior parietal and ventral premotor grasping neurons. J. Neurophysiol. 108, 1607–1619. 10.1152/jn.01158.2011 22745465

[B6] Bonini L. Serventi F. U. Simone L. Rozzi S. Ferrari P. F. Fogassi L. (2011). Grasping neurons of monkey parietal and premotor cortices encode action goals at distinct levels of abstraction during complex action sequences. J. Neurosci. 31, 5876–5886. 10.1523/JNEUROSCI.5186-10.2011 21490229 PMC6622840

[B7] Briggs G. G. Nebes R. D. (1975). Patterns of hand preference in a student population. Cortex 11, 230–238. 10.1016/S0010-9452(75)80005-0 1204363

[B8] Brown S. G. Roy E. A. Rohr L. E. Bryden P. J. (2006). Using hand performance measures to predict handedness. Laterality 11, 1–14. 10.1080/1357650054200000440 16414911

[B9] Bryden P. J. Huszczynski J. (2011). Under what conditions will right-handers use their left hand? The effects of object orientation, object location, arm position, and task complexity in preferential reaching. Laterality 16, 722–736. 10.1080/1357650X.2010.514344 21391107

[B10] Bryden P. J. Roy E. A. (2006). Preferential reaching across regions of hemispace in adults and children. Dev. Psychobiol. 48, 121–132. 10.1002/dev.20120 16489592

[B11] Calvert G. A. Bishop D. V. (1998). Quantifying hand preference using a behavioural continuum. Laterality 3, 255–268. 10.1080/713754307 15513088

[B12] Carlier M. Doyen A. L. Lamard C. (2006). Midline crossing: developmental trend from 3 to 10 years of age in a preferential card-reaching task. Brain Cogn. 61, 255–261. 10.1016/j.bandc.2006.01.007 16513237

[B13] Cavill S. Bryden P. (2003). Development of handedness: comparison of questionnaire and performance-based measures of preference. Brain Cogn. 53, 149–151. 10.1016/S0278-2626(03)00098-8 14607136

[B14] Corballis M. C. (1997). The genetics and evolution of handedness. Psychol. Rev. 104, 714–727. 10.1037/0033-295X.104.4.714 9337630

[B15] Coren S. (1995). Age and handedness: patterns of change in the population and sex differences become visible with increased statistical power. Can. J. Exp. Psychol. 49, 376–386. 10.1037/1196-1961.49.3.376 9183982

[B16] Coren S. Porac C. Duncan P. (1981). Lateral preference behaviors in preschool children and young adults. Child Dev. 52, 443–450 10.2307/1129160

[B17] Fagard J. Marks A. (2000). Unimanual and bimanual tasks and the assessment of handedness in toddlers. Dev. Sci. 3, 137–147 10.1111/1467-7687.00107

[B18] Flindall J. W. Gonzalez C. L. R. (2013). On the evolution of handedness: evidence for feeding biases. PLoS ONE 8:e78967. 10.1371/journal.pone.0078967 24236078 PMC3827312

[B19] Flindall J. W. Gonzalez C. L. R. (2014). Eating interrupted: the effect of intent on hand-to-mouth actions. J. Neurophysiol. 112, 2019–2025. 10.1152/jn.00295.2014 24990561

[B20] Flindall J. W. Stone K. D. Gonzalez C. L. R. (2014). Evidence for right-hand feeding biases in a left-handed population. Laterality. [Epub ahead of print]. 10.1080/1357650X.2014.961472 25256315

[B22] Fogassi L. Ferrari P. F. Gesierich B. Rozzi S. Chersi F. Rizzolatti G. (2005). Parietal lobe: from action organization to intention understanding. Science 308, 662–667. 10.1126/science.1106138 15860620

[B23] Gabbard C. Tapia M. Helbig C. R. (2003). Task complexity and limb selection in reaching. Int. J. Neurosci. 113, 143–152. 10.1080/00207450390161994 12751428

[B24] Geerts W. K. Einspieler C. Dibiasi J. Garzarolli B. Bos A. F. (2003). Development of manipulative hand movements during the second year of life. Early Hum. Dev. 75, 91–103. 10.1016/j.earlhumdev.2003.09.006 14652162

[B25] Gonzalez C. L. R. Goodale M. A. (2009). Hand preference for precision grasping predicts language lateralization. Neuropsychologia 47, 3182–3189. 10.1016/j.neuropsychologia.2009.07.019 19654015

[B26] Gonzalez C. L. R. Whitwell R. L. Morrissey B. Ganel T. Goodale M. A. (2007). Left handedness does not extend to visually guided precision grasping. Exp. Brain Res. 182, 275–279. 10.1007/s00221-007-1090-1 17717653

[B27] Gooderham S. E. Bryden P. J. (2014). Does your dominant hand become less dominant with time? The effects of aging and task complexity on hand selection. Dev. Psychobiol. 56, 537–546. 10.1002/dev.21123 23765799

[B28] Graziano M. S. (2006). The organization of behavioral repertoire in motor cortex. Annu. Rev. Neurosci. 29, 105–134. 10.1146/annurev.neuro.29.051605.112924 16776581

[B29] Graziano M. S. (2009). The Intelligent Movement Machine. Oxford: Oxford University Press 10.1093/acprof:oso/9780195326703.001.0001

[B30] Graziano M. S. Aflalo T. N. Cooke D. F. (2005). Arm movements evoked by electrical stimulation in the motor cortex of monkeys. J. Neurophysiol. 94, 4209–4223. 10.1152/jn.01303.2004 16120657

[B31] Graziano M. S. Cooke D. F. Taylor C. S. Moore T. (2004). Distribution of hand location in monkeys during spontaneous behavior. Exp. Brain Res. 155, 30–36. 10.1007/s00221-003-1701-4 15064882

[B32] Graziano M. S. Taylor C. S. Moore T. (2002). Complex movements evoked by microstimulation of precentral cortex. Neuron 34, 841–851. 10.1016/S0896-6273(02)00698-0 12062029

[B33] Hatta T. Ito Y. Matsuyama Y. Hasegawa Y. (2005). Lower-limb asymmetries in early and late middle age. Laterality 10, 267–277. 10.1080/13576500442000076 16019712

[B34] Hill E. L. Khanem F. (2009). The development of hand preference in children: the effect of task demands and links with manual dexterity. Brain Cogn. 71, 99–107. 10.1016/j.bandc.2009.04.006 19457603

[B35] Hugdahl K. Satz P. Mitrushina M. Miller E. N. (1993). Left-handedness and old age: do left-handers die earlier? Neuropsychologia 31, 325–333. 10.1016/0028-3932(93)90156-T 8502368

[B36] Hugdahl K. Zaucha K. Satz P. Mitrushina M. Miller E. N. (1996). Left-handedness and age: comparing writing/ drawing and other manual activities. Laterality 1, 177–183. 10.1080/713754239 15513035

[B37] Jacquet A. Y. Esseily R. Rider D. Fagard J. (2012). Handedness for grasping objects and declarative pointing: a longitudinal study. Dev. Psychobiol. 54, 36–46. 10.1002/dev.20572 21656764

[B38] Kalisch T. Wilimzig C. Kleibel N. Tegenthoff M. Dinse H. R. (2006). Age-related attenuation of dominant hand superiority. PLoS ONE 1:e90. 10.1371/journal.pone.0000090 17183722 PMC1762407

[B39] Kumar S. Misra I. Suman S. Suar D. Mandal M. K. (2010). Interrelationship of limb dominance and sensory function across age. Int. J. Neurosci. 120, 110–114. 10.3109/00207450903337168 20199202

[B40] Leconte P. Fagard J. (2004). Influence of object spatial location and task complexity on children's use of their preferred hand depending on their handedness consistency. Dev. Psychobiol. 45, 51–58. 10.1002/dev.20019 15340974

[B41] Mamolo C. M. Roy E. A. Bryden P. J. Rohr L. E. (2004). The effects of skill demands and object position on the distribution of preferred hand reaches. Brain Cogn. 55, 349–351. 10.1016/j.bandc.2004.02.041 15177810

[B42] Mamolo C. M. Roy E. A. Rohr L. E. Bryden P. J. (2006). Reaching patterns across working space: the effects of handedness, task demands, and comfort levels. Laterality 11, 465–492. 10.1080/13576500600775692 16882557

[B43] McManus I. Sik G. Cole D. Mellon A. Wong J. Kloss J. (1988). The development of handedness in children. Br. J. Dev. Psychol. 6, 257–273 10.1111/j.2044-835X.1988.tb01099.x

[B44] Michel G. F. (1981). Right-handedness: a consequence of infant supine head-orientation preference? Science 212, 685–687. 10.1126/science.7221558 7221558

[B45] Michel G. F. Nelson E. L. Babik I. Campbell J. M. Marcinowski E. C. (2013). Multiple trajectories in the developmental psychobiology of human handedness. Adv. Child Dev. Behav. 45, 227–260. 10.1016/B978-0-12-397946-9.00009-9 23865118

[B46] Oldfield R. C. (1971). The assessment and analysis of handedness: the Edinburgh inventory. Neuropsychologia 9, 97–113. 10.1016/0028-3932(71)90067-4 5146491

[B47] Porac C. (1993). Are age trends in adult hand preference best explained by developmental shifts or generational differences? Can. J. Exp. Psychol. 47, 697–713. 10.1037/h0078873 8124292

[B48] Porac C. Coren S. Duncan P. (1980). Life-span age trends in laterality. J. Gerontol. 35, 715–721. 10.1093/geronj/35.5.715 7430568

[B49] Porac C. Friesen I. C. (2000). Hand preference side and its relation to hand preference switch history among old and oldest-old adults. Dev. Neuropsychol. 17, 225–239. 10.1207/S15326942DN1702_05 10955204

[B50] Porac C. Searleman A. (2002). The effects of hand preference side and hand preference switch history on measures of psychological and physical well-being and cognitive performance in a sample of older adult right-and left-handers. Neuropsychologia 40, 2074–2083. 10.1016/S0028-3932(02)00058-1 12208004

[B51] Porac C. Searleman A. (2006). The relationship between hand preference consistency, health, and accidents in a sample of adults over the age of 65 years. Laterality 11, 405–414. 10.1080/13576500600677823 16882554

[B52] Rat-Fischer L. O'Regan J. K. Fagard J. (2013). Handedness in infants' tool use. Dev. Psychobiol. 55, 860–868. 10.1002/dev.21078 22949283

[B53] Sacrey L. A. Arnold B. Whishaw I. Q. Gonzalez C. L. R. (2013). Precocious hand use preference in reach-to-eat behavior versus manual construction in 1- to 5-year-old children. Dev. Psychobiol. 55, 902–911. 10.1002/dev.21083 23129422

[B54] Sacrey L. A. Karl J. M. Whishaw I. Q. (2012). Development of rotational movements, hand shaping, and accuracy in advance and withdrawal for the reach-to-eat movement in human infants aged 6-12 months. Infant Behav. Dev. 35, 543–560. 10.1016/j.infbeh.2012.05.006 22728335

[B55] Scharoun S. M. Bryden P. J. (2014). Hand preference, performance abilities, and hand selection in children. Front. Psychol. 5:82. 10.3389/fpsyg.2014.00082 24600414 PMC3927078

[B56] Steenhuis R. E. Bryden M. P. (1989). Different dimensions of hand preference that relate to skilled and unskilled activities. Cortex 25, 289–304. 10.1016/S0010-9452(89)80044-9 2758854

[B57] Stone K. D. Bryant D. C. Gonzalez C. L. R. (2013). Hand use for grasping in a bimanual task: evidence for different roles? Exp. Brain Res. 224, 455–467. 10.1007/s00221-012-3325-z 23161156

[B58] Stone K. D. Gonzalez C. L. R. (2014). Grasping with the eyes of your hands: hapsis and vision modulate hand preference. Exp. Brain Res. 232, 385–393. 10.1007/s00221-013-3746-3 24162864

[B59] Tapley S. M. Bryden M. P. (1985). A group test for the assessment of performance between the hands. Neuropsychologia 23, 215–221. 10.1016/0028-3932(85)90105-8 4000456

[B60] Weller M. P. Latimer-Sayer D. T. (1985). Increasing right hand dominance with age on a motor skill task. Psychol. Med. 15, 867–872. 10.1017/S0033291700005109 4080890

